# Impaired intracellular trafficking defines early Parkinson's disease

**DOI:** 10.1016/j.tins.2014.12.009

**Published:** 2015-03

**Authors:** Benjamin H.M. Hunn, Stephanie J. Cragg, J. Paul Bolam, Maria-Grazia Spillantini, Richard Wade-Martins

**Affiliations:** 1 Oxford Parkinson's Disease Centre, University of Oxford, South Parks Road, Oxford OX1 3QX, UK; 2 Department of Physiology, Anatomy and Genetics, University of Oxford, South Parks Road, Oxford OX1 3QX, UK; 3 Medical Research Council Anatomical Neuropharmacology Unit, Department of Pharmacology, University of Oxford, Mansfield Road, Oxford OX1 3QT, UK; 4 Department of Clinical Neurosciences, University of Cambridge, The Clifford Allbutt Building, Hills Road, Cambridge CB2 0QH, UK

**Keywords:** Parkinson's disease, cell trafficking, Tau, α-synuclein

## Abstract

Parkinson's disease (PD) is an insidious and incurable neurodegenerative disease, and represents a significant cost to individuals, carers, and ageing societies. It is defined at post-mortem by the loss of dopamine neurons in the substantia nigra together with the presence of Lewy bodies and Lewy neurites. We examine here the role of α-synuclein and other cellular transport proteins implicated in PD and how their aberrant activity may be compounded by the unique anatomy of the dopaminergic neuron. This review uses multiple lines of evidence from genetic studies, human tissue, induced pluripotent stem cells, and refined animal models to argue that prodromal PD can be defined as a disease of impaired intracellular trafficking. Dysfunction of the dopaminergic synapse heralds trafficking impairment.

## Highlights

• The dopamine neurons that die in Parkinson's disease are anatomically complex.
• Cell trafficking is impaired by the key pathogenic protein in Parkinson's, α-synuclein.
• Anatomical complexity means that dopamine neurons are susceptible to trafficking deficits.
• Familial Parkinson's genes are involved in intracellular trafficking.

## Early PD: a traffic jam

PD is a common neurodegenerative disease characterised by insidious deterioration of motor control, often associated with mood, sleep, and cognitive disturbances [1]. Over 1% of all people over the age of 65 suffer from PD [2]. Similarly to other neurodegenerative diseases, age is a key risk factor and by 2030, an estimated 9 million people worldwide will be living with PD [3]. PD carries a significant economic cost, including direct and indirect health care costs, and lost productivity [4], estimated annually at £500 million per year in the UK, and $6 billion in the USA 5, 6. PD is pathologically characterised by the loss of midbrain dopamine (DA; see Glossary) neurons, and the development of Lewy bodies and Lewy neurites that are predominantly composed of the protein α-synuclein [7].

The aim of this review is to integrate lines of evidence from human tissue, human iPSCs, refined animal models, and genetic studies that have suggested early PD pathogenesis is likely to be a consequence of, and be defined by, impaired intracellular trafficking. We discuss how dysfunction of key intracellular trafficking proteins, together with the large demand on the intracellular trafficking system that the massive axonal arbor of midbrain DA neurons impose, underlie the pathogenesis of PD.

## A complex road-map: the DA neuron

**Figure 1:**
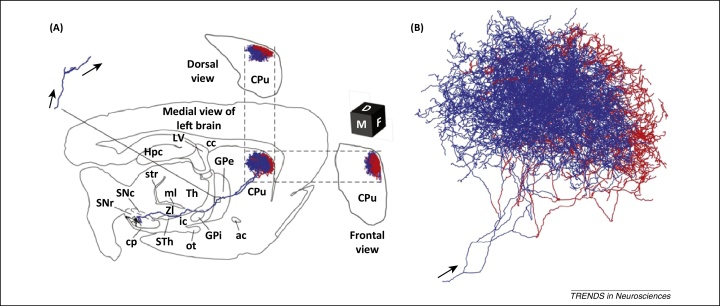
Complex axonal arborisation of midbrain dopaminergic neurons. **(A)** Medial, dorsal, and frontal reconstructions of the axonal projections of midbrain dopaminergic neurons generated using a GFP protein targeting neuronal membranes. Red (striosome) and blue (matrix) lines indicate the striatal compartments in which axonal fibres are located. **(B)** Striatal arborisation of a typical midbrain dopaminergic neuron projected onto the parasagittal plane. Figures adapted from Matsuda *et al.*[13] with permission from the Society for Neuroscience. Abbreviations: ac, anterior commissure; cc, corpus callosum; cp, cerebral peduncle; CPu, caudate-putamen (neostriatum); GPe, external segment of the globus pallidus; GPi, globus pallidus interna; Hpc, hippocampus; ic, internal capsule; LV, lateral ventricle; ml, medial lemniscus; ot, optic tract; SNc, substantia nigra pars compacta; SNr, substantia nigra pars reticulata; STh, subthalamic nucleus; str, superior thalamic radiation; Th, thalamus; ZI, zona incerta.

The motor manifestations of PD are largely due to degeneration of DA neurons in the substantia nigra pars compacta (SNc), and to a lesser extent the ventral tegmental area (VTA) and other midbrain regions. These nigral neurons project via the mesostriatal pathway to the striatum (caudate-putamen). In the striatum, the axons of DA neurons branch extensively giving rise to a dense lattice that provides non-selective DA innervation of the principal efferents of the striatum, the medium spiny neurons (Figure 1) 8, 9. Neuropathological studies have clearly demonstrated that the key aspects that define neuronal susceptibility in PD are the axonal length, axonal calibre, and the degree of myelination [10]. For example, cortical motor neurons that have relatively long processes but high-calibre axons that are heavily myelinated are relatively protected in PD, whereas most of the subcortical nuclei (chiefly the SNc, but also the magnocellular nuclei of the basal forebrain, hypothalamic tuberomamillary nucleus) have thin axons, are lightly myelinated, and develop Lewy pathology.

A close examination of the neuroanatomical and physiological characteristics of SNc DA neurons helps us to understand why these neurons are most severely affected in PD. Quantitative anatomical data lead to an estimate that each SNc DA neuron in the rat gives rise to 100 000–250 000 synapses at the level of the striatum [11]. The extraordinary number of synapses formed by each DA neuron is put into context by considering other neuronal types in the rat: striatal spiny neurons form ∼300 synapses, striatal inhibitory interneurons give rise to ∼5 000 synapses, and on average there are ∼10 000 synapses per cortical neuron [12]. An elegant study in the rat has corroborated these findings by labelling individual DA neurons in the SNc in their entirety, allowing the immense nature of their axonal arbors to be visualised and quantified (Figure 1) [13]. The average total length of the axon of a rat SNc DA neuron was estimated at ∼50 cm and the average volume occupied by the axonal arbor at about ∼0.5 mm^3^. Extrapolation of these findings to the human brain, the striatal volume of which is about 300-fold greater than in the rat, but with only ∼30-fold more SNc neurons to provide the DA innervation, indicates that an individual human DA neuron provides 10-fold more innervation than does a rat DA neuron. This suggests that each human SNc DA neuron gives rise to between 1 and 2.5 million synapses in the striatum, with a total axonal length in excess of 4 m [11]. Trafficking of vesicles and organelles, including synaptic vesicles, mitochondria, and ribosomes, throughout the cell is dependent on transport along microtubules (reviewed by Hancock [14]). The requirement for cellular trafficking machinery in human SNc DA neurons is far in excess of other neuron types, and means that any impairment of cellular trafficking preferentially affects SNc DA neurons. Cellular trafficking places energy demands on the cell because the predominant motor proteins, kinesin and dynein, are powered by hydrolysis of ATP; one molecule of ATP is required for 8 nm of cellular travel [15]. In addition, computational analysis [16] reveals that such a large and complex axonal arborisation incurs a disproportionately high energy-cost for action potential propagation and recovery of membrane potential.

Other factors collude to increase the vulnerability of SNc DA neurons. SNc DA neurons compared to adjacent VTA neurons have elevated somatodendritic calcium entry associated with intrinsic pacemaking currents [17], while their axons use different complements of calcium channels with evidence for differential calcium handling [66]. Some studies also describe DA and its metabolites as neurotoxic, and there is also the potential for deleterious DA modification of α-synuclein [18].

In summary, the massive highly-tortuous axonal arborisation and large synapse number of vulnerable human SNc DA neurons imposes a disproportionately large burden on the machinery for cellular transport and synaptic release, and may explain why these neurons are preferentially susceptible in PD, despite the wider distribution of key pathogenic proteins such as α-synuclein.

## SNAREd in traffic: α-synuclein physiology and pathology

**Figure 2:**
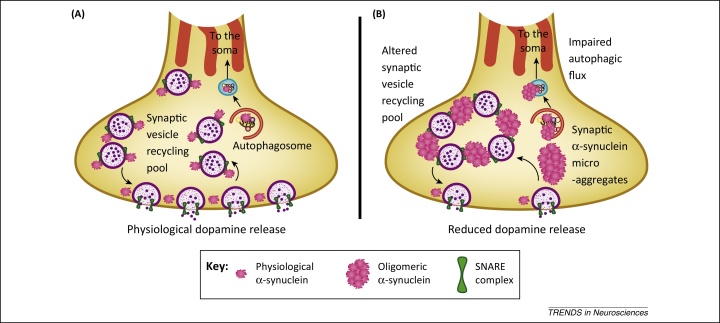
Synaptic dystrophy in Parkinson's disease. **(A)** Tight regulation of synaptic dopamine (DA) release is achieved by SNARE-mediated vesicle docking, together with chaperone molecules cysteine string protein-α (CSPα) and the synucleins. Autophagosomes are able to leave the synapse, hence preventing protein accumulation. **(B)** α-Synuclein disrupts synaptic physiology. The presence of oligomeric α-synuclein impairs SNARE function, decreasing intervesicular space, reducing the number of synaptic vesicles, and impairing DA release. Autophagosomes fuse with lysosomes in the cell body, but are prevented from leaving the synapse, and *MAPT* haplotype differences may contribute to impaired axonal transport of autophagosomes.

**Figure 3:**
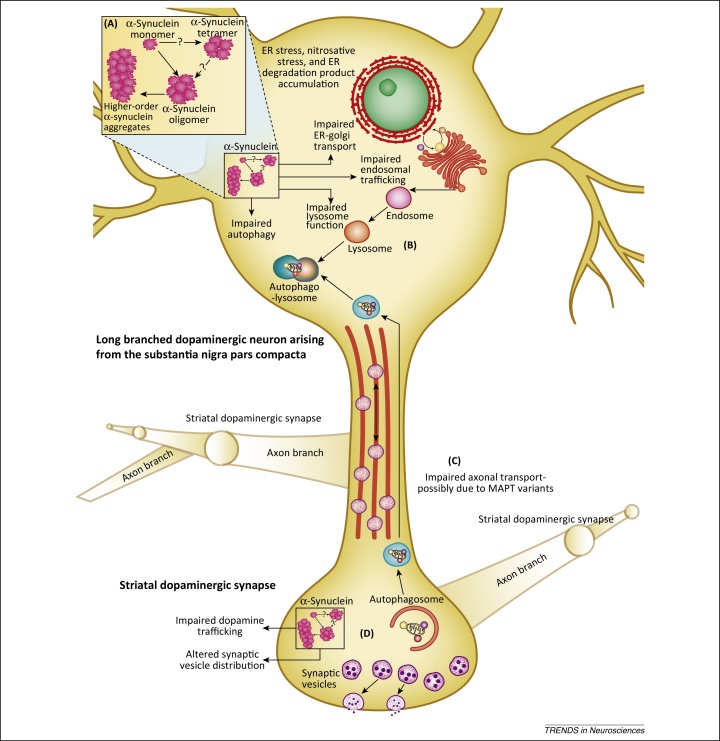
Intracellular trafficking is impaired in Parkinson's disease (PD). **(A)** The pathological and physiological species of α-synuclein remain unknown; however, increasing evidence suggests that oligomers are responsible for the majority of the early deleterious effects. **(B)** Recent findings demonstrate that α-synuclein impairs key events in the soma, such as ER–Golgi trafficking, endosomal trafficking, and autophagolysosome formation. **(C)** The most significant genetic risk factor for sporadic PD is the gene for the microtubule-associated protein tau (*MAPT*). The key function of the tau protein is to regulate microtubule stability, allowing efficient axonal transport, thus providing a mechanism by which *MAPT* variants confer PD susceptibility. Increased α-synuclein also impairs axonal transport. **(D)** At the synapse, α-synuclein disturbs DA trafficking and synaptic vesicle distribution. Importantly, synaptic autophagosomes must be trafficked to the soma for protein degradation.

Multiple independent avenues of research have implicated the protein α-synuclein in the pathogenesis of PD, including the presence of α-synuclein in Lewy bodies, inherited cases of PD caused by mutations in the gene encoding α-synuclein (*SNCA*), and genome-wide association studies linking sporadic PD and *SNCA* 19, 20, 21. Despite controversy regarding the physiological state of α-synuclein (Box 1), increasing evidence suggests that the physiological role of α-synuclein is to regulate exocytosis of neurotransmitter at the synapse. α-Synuclein acts as a chaperone for soluble *N*-ethylmaleimide-sensitive factor attachment protein receptor (SNARE) complexes. SNARE complexes mediate membrane fusion to allow synaptic vesicle exocytosis, after which they rapidly disassociate to an unfolded state (Figure 2). Disassociated SNARE proteins are prone to misfolding and non-specific interactions, and thus there are four chaperone proteins to safeguard SNARE proteins at the synapse: cysteine string protein-α (CSPα) and the synucleins [22]. The three members of the synuclein family, α-, β-, and γ-synuclein, are highly homologous proteins that bind to phospholipids as α-helices. Studies in combinatorial knockout mice have demonstrated some functional overlap in the role of the synucleins, and indicated that the synucleins directly or indirectly regulate DA releasability, with synuclein deletions increasing evoked DA release 23, 24. Knockout of CSPα is neurotoxic, and overexpression of α-synuclein can protect against this effect 22, 25. However, despite this specific neuroprotective role, α-synuclein also promotes neurodegeneration. It is likely that environmental insults, age-related impairment of autophagy, changes in the SNCA gene, and other factors together precipitate α-synuclein accumulation, prompting oligomerisation 26, 27, 28, 29. The overwhelming body of evidence now indicates that accumulation of α-synuclein, and possibly oligomeric species in the absence of insoluble aggregates, give rise to deleterious effects in DA neurons (Figure 3) 30, 31, 32, 33.

The physiological localization of α-synuclein, predominantly at the presynaptic terminal, is associated with the major early pathological manifestations of PD: impaired DA release and synaptic dystrophy. This is demonstrated by a new mouse model of PD, a bacterial artificial chromosome (BAC) transgenic that incorporates the human *SNCA* genomic locus with flanking regulatory elements, and expresses human α-synuclein in a spatially- and temporally-relevant manner similar to the physiological distribution [34]. This mouse model exhibits reduced DA transmission in dorsal but not ventral striatum, as well as alterations in the distribution of synaptic vesicles that precede α-synuclein accumulation, a motor phenotype, and neuron loss [34]. Analysis of synaptic vesicles in dorsal striatal DA axons of this model revealed that compromised DA transmission correlates with decreased intervesicle distance, indicating increased clustering, consistent with observations that α-synuclein attenuates the mobility, intersynaptic dispersion, and size of the synaptic vesicle recycling pool 35, 36. This builds on other work which found compromised DA transmission in PD models produced by injection of viral vectors overexpressing α-synuclein in the rat [37] and in transgenic mice expressing the Ala30Pro [38] or Ala53Thr [39] mutated versions of human α-synuclein. Expression of a truncated, aggressively aggregating form of α-synuclein leads to a reduction in DA release that is accompanied by a redistribution of SNARE proteins that parallels changes seen in PD patients [40]. Furthermore, truncated α-synuclein causes dystrophy at the level of the synapse, before overtly affecting the cell body or dendrites. This is consistent with biochemical studies demonstrating that α-synuclein inhibits SNARE-mediated vesicle fusion [41]. Newly defined insoluble α-synuclein ‘microaggregates’ have been shown to form in presynaptic DA terminals, adding weight to the argument that toxic aggregated species can disrupt SNARE function at synapses (Figure 2) 40, 42.

Taken together, these findings suggest that impaired intracellular trafficking in PD disturbs the regulation of the DA synapse, particularly in dorsal striatum, which represents the ‘canary in the coal mine’ of PD pathogenesis. The interplay between structure and pathogenicity probably plays a key role here; the need to maintain a synapse distant from the cell body may isolate the DA synapse to an extent not seen in other neurons. Advances in synaptic imaging have allowed quantification of synaptic structure through super-resolution fluorescence and electron microscopy, quantitative immunoblotting, and mass spectrometry [43]. By applying the same techniques to striatal DA synapses from healthy and diseased brains we might increase our understanding of how this synapse is first altered in PD (Box 2).

## α-Synuclein impedes the endoplasmic reticulum (ER)–Golgi roundabout

The development of human iPSCs has prompted a new raft of data from PD patient-derived DA neurons [44]. iPSCs are pluripotent stem cells derived from adult human tissues which can be differentiated into multiple cell types, including DA neurons [45]. iPSCs generated from patients harbouring α-synuclein point mutations and triplications allow interrogation of downstream effects of α-synuclein on neuronal biology 46, 47. Although iPSCs are particularly well-suited to determining early disease-related phenotypes, it remains unclear how long iPSCs should be aged to develop features associated with late-stage disease. The key phenotype found in these neurons is accumulation of ER-associated degradation (ERAD) products and nitrosative stress, confirming earlier findings in *Drosophila* 46, 47, 48. Ryan *et al.* described nitrosative stress in iPSC neurons harbouring the A53T α-synuclein mutation, and linked this to mitochondrial dysfunction and neuronal apoptosis [49]. In a yeast-based ER–Golgi model, α-synuclein markedly attenuates vesicle docking and fusion, but without affecting vesicle formation 48, 50. Further studies in yeast identified a novel agent, *N*-aryl benzimidazole (NAB), that, when used in iPSC neurons, ameliorated the PD phenotype by stimulating endosomal transport, suggesting that intracellular transport deficits are key to early PD 46, 47. NAB exerts its effect via Nedd4, a ubiquitin ligase that has diverse roles in neuronal transport through its ability to regulate ubiquitin-dependent trafficking, and has been implicated in α-synuclein degradation [51]. The advent of PD-patient derived iPSC neurons has yielded evidence that dysfunction in ER–Golgi complex trafficking is a major factor in disease progression 48, 52.

Parallels are found in other trafficking pathways, most notably the autophagy-lysosome pathway, which is also impaired in early PD. Decressac and colleagues showed that α-synuclein overexpression in rat causes a decline in lysosomal function and that associated neurodegeneration can be prevented by overexpressing transcription factor EB (TFEB), a regulator of the autophagy–lysosome pathway [53]. An important link between trafficking impairment at the levels of the synapse and the soma is the observation that α-synuclein-induced disruption of ER–Golgi trafficking occurs through direct interaction between α-synuclein and ER–Golgi SNARE complexes [52]. Several other key membrane fusion pathways with relevance to PD rely on SNARE machinery, including the autophagy pathway [54], and there is potential for these to be disrupted by accumulation of α-synuclein.

## Tau-rafficking: the chromosome 17 H1 haplotype and PD

One of the genetic regions most significantly associated with sporadic PD in genome-wide association studies is located on chromosome 17q21 19, 55. This region has two haplotypes, designated H1 and H2. The less-common H2 haplotype results from a 970 kb inversion within the H1 genetic sequence [56]. The 17q21 H1 haplotype has been found to associate with PD and the atypical parkinsonian syndromes progressive supranuclear palsy (PSP) and corticobasal degeneration (CBD) in large-cohort studies [57]. In contrast to the deleterious effects of H1, the H2 haplotype is neuroprotective [56]. The question as to how the H1 haplotype contributes to disease susceptibility has been extensively investigated [58]. Multiple genes are associated with the H1 haplotype which could potentially confer risk for PD. The gene for which there is most evidence for PD-risk associated with the H1 haplotype is the microtubule-associated protein tau (*MAPT*) locus. Another key gene with a role in cellular trafficking that may help to explain the PD risk arising from genetic variation in the 17q21 region is *NSF*, which encodes *N*-ethylmaleimide-sensitive factor, a protein that disassembles SNARE complexes and regulates vesicular transport [59]. The effect of the H1 and H2 haplotypes on NSF expression and function are not well understood and would benefit from investigation. Other genes associated with the H1 haplotype include *IMP5* and *CRHR1* [58].

**Figure 4:**
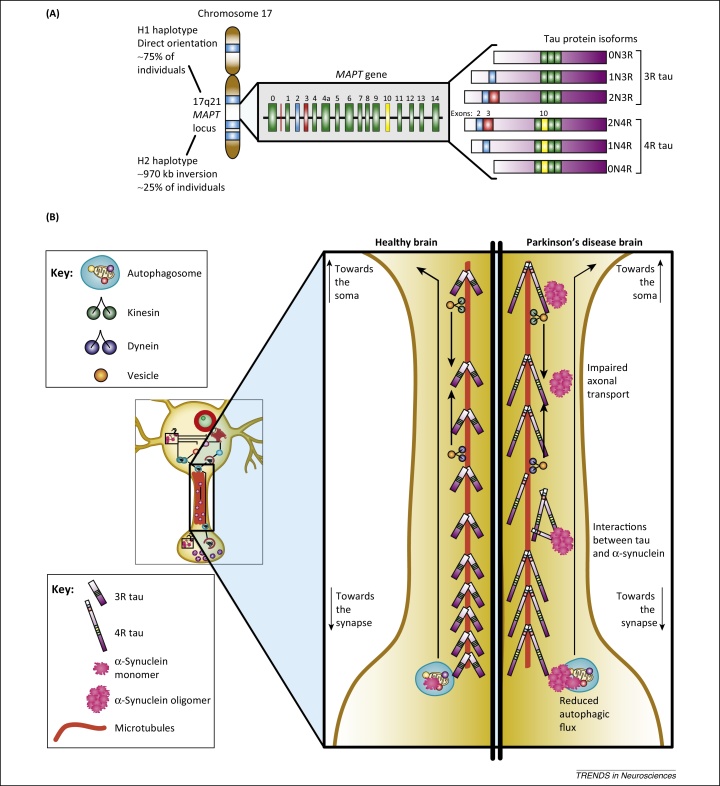
Relationship of tau with axonal transport. **(A)** Differential expression of tau protein isoforms arise from translation of *MAPT* splice variants. The *MAPT* H1 haplotype is directly oriented, whereas the H2 haplotype is due to an inverted sequence of approximately 970 kb. Alternative splicing events at exons 2 and 3 generate tau protein with zero (0N), one (1N), or two (2N) N-terminal repeats. Tau protein with three (3R) or four (4R) tandem repeats is generated from alternative splicing at exon 10. H1 causes relatively increased expression of exon 10, and therefore 4R tau. H2 causes increased exon 3 expression, and therefore 2N tau. Figure adapted from Wade-Martins [60]. **(B)** Comparison of axonal transport in healthy and Parkinson's disease (PD) dopaminergic neurons. In healthy brain, an increasing tau gradient from proximal to distal helps to drive axonal transport mediated by transport proteins, particularly kinesin and dynein. In the PD brain, risk-associated *MAPT* variants increase 4R tau expression, which is more prone to aggregation. α-Synuclein impairs axonal transport. Autophagosomes must be cleared from the synapse to the soma to fuse with the lysosome for protein degradation, but this clearance is reduced as a result of axonal trafficking impairment.

*MAPT* encodes the protein tau, which is highly expressed in neurons and has several functions such as microtubule stabilisation and elongation, and axonal transport [60]. The impact of *MAPT* variants on axonal trafficking, and the unique architecture of the DA neuron, may help to explain the association between *MAPT* and PD. *MAPT* encodes six tau isoforms, which are generated by alternative splicing of exons 2, 3, and 10 (Figure 4). Alternative splicing events at exons 2 and 3 generate tau protein with zero (0N), one (1N), or two (2N) N-terminal repeats. Tau isoforms with three (3R) or four (4R) tandem repeats are generated from alternative splicing of exon 10 [58]. There is strong evidence that alternative splicing of exon 10 is under haplotype-specific control 61, 62. Increased 4R:3R tau ratio driven by the H1 haplotype has been reported in several experimental contexts and in human brain mapping 63, 64. Zhong and colleagues have recently used refined biochemical techniques to examine the effect of tau splice variants on aggregation propensity, demonstrating that 4R tau has more rapid aggregation kinetics than 3R tau [65]. Chu *et al.* have demonstrated that there is a reduction in axonal transport proteins, particularly kinesin, in sporadic PD and in an α-synuclein-based rat model [67]. Tau interacts with kinesin and dynein, and shorter forms of tau more strongly inhibit axonal transport (Figure 4) 68, 69. Impaired axonal trafficking gains importance when the need to traffic aggregated proteins, including α-synuclein, retrogradely to the soma for clearance by the autophagy–lysosome pathway is considered [70]. Mitochondria and RNA also depend on microtubule-associated transport for correct synaptic localisation. Notably, patients with mutations in the gene encoding dynactin, a protein that interacts with dynein and kinesin, develop Perry syndrome, an atypical inherited form of parkinsonism [71]. There is also the potential for interaction between tau and α-synuclein. Studies in a double transgenic *Drosophila* model co-expressing tau and α-synuclein have shown ubiquitin-positive aggregates of both proteins and additive impairment of axonal transport associated with cytoskeletal disorganization and synaptic dystrophy [72].

## LRRKing about in traffic

*LRRK2* encodes for leucine-rich repeat kinase 2 (LRRK2), a large multidomain protein that encompasses a GTPase domain, a kinase domain, and three other putative protein interaction domains. Mutations in *LRRK2* were first causally associated with autosomal dominant PD in 2004, and it was subsequently found that variation in *LRRK2* is associated with up to 40% of sporadic PD cases in some populations [73]. The roles of LRRK2 in the cell remain unclear; however, there is increasing evidence that it facilitates vesicle recycling, including at the synapse and associated with autophagy. Piccoli *et al.* observed that RNA-mediated silencing of LRRK2 in cultured cortical neurons caused a reduced number of docked vesicles at the presynapse, but a greater number in the synaptic reserve pool [74]. This correlated with altered postsynaptic currents with paired stimulations [74]. Matta and colleagues have shown that LRRK2 regulates EndoA, an evolutionarily conserved synaptic protein that drives vesicle formation [75]. Transgenic mice expressing both wild type and mutant human LRRK2 have reduced basal DA release, and mice with the most common LRRK2 mutation, G2019S, demonstrate age-related alterations in tau phosphorylation [76].

Others have suggested roles for LRRK2 in tau phosphorylation, cytoskeletal processes, and cellular trafficking which are of relevance given the large axonal arbor of DA neurons [77]. Recent evidence from an unbiased protein-interaction study has demonstrated that LRRK2 interacts with proteins encoded by two other candidate genes for sporadic PD susceptibility: rab-7-like protein 1 (*RAB7L1*/*RAB29*) and cyclin-G-associated kinase (*GAK*) [78]. This confirms previous work that identified Rab7L1 as a LRRK2 binding partner [79]. The LRRK2–Rab7L1–GAK complex promotes clearance of Golgi-derived vesicles through the autophagy–lysosome pathway, consistent with previous experiments demonstrating that LRRK2 activity regulates autophagy [80]. Notably, the *RAB7L1* locus modifies *LRRK2*-associated risk in four genome-wide association study (GWAS) cohorts, underscoring the potential therapeutic importance of LRRK2–Rab7L1 interactions [79]. These new findings indicate that several genetic loci significantly associated with sporadic PD represent one biological complex with a crucial role in PD pathogenesis and cellular trafficking.

## Familial PD genes: traffic wardens

Inherited forms of PD constitute 5–10% of all cases and remain important not only in our understanding of the disease in these individuals, but also by informing lines of inquiry in the more-complex pathogenesis of sporadic PD. Most confirmed causes of familial PD feature mutations in cellular trafficking proteins (see Table 1). The most common causes of familial PD are mutations in parkin (*PARK2*), which encodes a ubiquitin ligase, and PTEN-induced kinase 1 (*PINK1*). Both participate in the degradation of mitochondria by autophagy (mitophagy), and there is an emerging role of PINK1 in regulating mitochondrial trafficking 81, 82. *GBA* encodes glucocerebrosidase, a lysosomal hydrolase that cleaves the β-glucosyl linkage of glucosylsphingosine and glucosylceramide. Patients who suffer from Gaucher's disease, a lysosomal storage disease, are homozygous for mutations in *GBA*. A subset of patients with Gaucher's disease exhibit parkinsonian symptoms. Patients who carry heterozygous *GBA* mutations have an increased risk of developing PD, such that some authors consider *GBA* a dominant causal PD gene with reduced penetrance 83, 84. Reduced glucocerebrosidase activity is correlated with α-synuclein accumulation in sporadic PD [85]. Mazzulli *et al.* have recently shown in human Gaucher's disease iPSC and α-synuclein mouse-model neurons that glucocerebrosidase mediates proteolytic breakdown of α-synuclein, and, interestingly, tau [86]. Subsequent α-synuclein aggregation, in turn, caused reduced glucocerebrosidase activity, forming a possible positive-feedback loop in synucleinopathy pathogenesis.

Recent descriptions of new genes associated with monogenic PD have underscored the importance of cellular trafficking pathways in disease pathogenesis. Missense mutations in the vacuolar sorting protein 35 gene (VPS35) cause autosomal dominant PD 87, 88. *VPS35* encodes a subunit of the retromer complex, which is involved in membrane trafficking between endosomes and the *trans*-Golgi network, and more recently *VPS35* mutations have been shown to impair autophagy and cause SNc neurodegeneration when expressed in rats 89, 90. In the past year, mutations in two genes, *SYNJ1* and *DNAJC6*, have been identified in cases of juvenile parkinsonism 91, 92, 93, 94. *SYNJ1* encodes synatptojanin-1, a phosphoinositide phosphatase protein that is presynaptically enriched and has a role in synaptic vesicle endocytosis [95]. Similarly, *DNAJC6* encodes auxilin, a protein that is selectively expressed in neurons and allows clathrin-mediated Golgi–lysosome trafficking and synaptic vesicle endocytosis [93].

## Taking the wrong exit: the potential for spreading pathology in PD

Prions are protein aggregates that lack nucleic acid and can be transmitted from neuron-to-neuron, with the prion acting as a template for further production of misfolded proteins in healthy neurons. Disease can thus be propagated throughout the nervous system. There are several relatively rare human prion diseases, including Creutzfeldt–Jakob disease, fatal familial insomnia, and kuru. There are now emerging bodies of thought that more common neurodegenerative diseases, such as Alzheimer's disease and PD, may spread through ‘prion-like’ mechanisms. Prion-like spread is consistent with the stepwise progression of PD pathology throughout the brain proposed by Braak *et al.* – from the olfactory bulb, through the midbrain, to the cortex [96]. A series of clinical and laboratory findings have provided support for the hypothesis that PD represents a prion-like disorder. A key discovery was the post-mortem finding in three PD patients that Lewy bodies developed in foetal neural grafts 11 to 16 years after transplantation 97, 98. This contrasted with an earlier report of an autopsy of a similar patient performed 18 months following transplantation of foetal tissue, where survival of transplanted neurons and dense innervation of the striatum occurred but with no overt pathology [99]. These findings prompted several experiments in rodents to elucidate the nature of spreading α-synuclein pathology. Luk and colleagues showed that injection of synthetic α-synuclein into the dorsal striatum of wild type mice led to spreading Lewy pathology, including to the substantia nigra, and associated motor deficits [100]. α-Synuclein transfer to cultured dopaminergic neurons grafted into α-synuclein transgenic mice has also been observed, recapitulating the clinical findings 101, 102. While the existence of spreading PD pathology is now well documented, the mechanisms underlying the spreading pathology of PD are in the early stages of investigation. Exocytosis of α-synuclein has been observed [103] and more recently, multiple *in vitro* studies have demonstrated α-synuclein secretion in exosomes 104, 105. Impairing cell trafficking in the autophagy-lysosome pathway prompts exosomal release of α-synuclein 104, 105. Likewise, mutations in the *ATP13A2* gene, which encodes a lysosomal membrane transporter, and which cause the familial parkinsonian syndrome Kufor–Rakeb, also prompt exosomal secretion of α-synuclein [106].

## The long road home

The ultimate test of any hypothesis of disease causation is that removal of the proposed disease-causing agent leads to disease remission or stabilisation. Intracellular trafficking appears to be impaired early in the natural history of PD. Strategies to increase intracellular trafficking are yet to be developed for PD. One possible area for exploration is stimulation of the autophagy pathway, which has previously been shown to promote longevity and shown promise in treatment of other neurodegenerative proteinopathies [107]. However, currently available autophagy inducers, chiefly rapamycin, are limited in their utility because of other effects, namely immunosuppression. Strategies to reduce α-synuclein may improve intracellular trafficking and possibly prevent the spread of PD. Active and passive α-synuclein immunisation strategies have demonstrated efficacy in animal models, and at least one α-synuclein vaccine is in clinical trials [108]. It has been demonstrated *in vitro* that antibodies to α-synuclein prevent exosomal neuron-to-neuron transmission, and thus may halt disease progression [104]. Investigators have also described the possibility of using exogenous exosomes containing RNA sequences that reduce α-synuclein expression [109]. Any data relating to PD treatment must be interpreted in the light of the morphological characteristics of DA neurons as a contributory factor to their susceptibility to degenerate in PD – a factor which may prove to be limiting as therapeutics progress. The recent advances in PD neuron phenotyping reviewed here have raised the possibility of classifying diseases based on early disease manifestations rather than on traditional post-mortem pathology. PD may come to be recognised primarily as a disease of impaired intracellular trafficking, a process on which dopaminergic neurons are exquisitely dependent, and hence acutely vulnerable to its disturbance.

## Figures and Tables

**Figure I:**
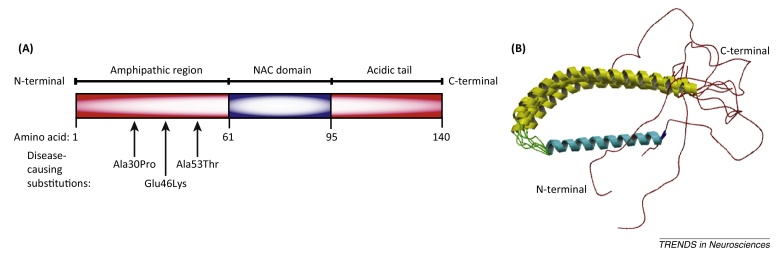
The α-synuclein protein, in schematic form **(A)** and its micelle-bound structure **(B)** solved using nuclear magnetic and electron paramagnetic resonance (taken from the Protein Databank, ID: 1XQ8 [115]). It is divided into three domains, the amphipathic region, the non-amyloid-β component of Alzheimer's disease amyloid (NAC) domain, and the C-terminal acidic tail.

**Table 1:** Relationship between confirmed familial PD genes and intracellular trafficking

Gene	Inheritance^a^	Clinicopathological phenotype	Function of the gene product	Refs
*SNCA*	AD	Early-onset, severe PD with Lewy Bodies	Interacts with SNARE proteins. Regulates neurotransmitter release and long-term synaptic homeostasis. Presynaptically enriched	20, 34
*LRRK2*	AD	Typical, late-onset PD with Lewy bodies	Has GTPase and kinase domains. Important roles in synaptic mechanics, endocytosis, and autophagy pathways	75, 80
*GBA*	AD	Typical, late-onset PD with Lewy bodies	Cleaves glucocerebroside during glycolipid metabolism. Interacts with α-synuclein in the lysosome	[86]
*VPS35*	AD	Typical, late-onset PD with unknown pathology	Role in endosome–Golgi complex trafficking; mutations impair autophagy.	[89]
*Parkin*	AR	Early-onset PD with slow progression and no Lewy bodies in most cases	Ubiquitin ligase that catalyses ubiquitin transfer to mitochondria for mitophagy	[82]
*PINK1*	AR	Early-onset PD with slow progression and Lewy body pathology	Has an essential role in mitophagy, and an emerging role in mitochondrial trafficking	81, 116
*DJ-1*	AR	Early-onset PD with slow progression and unknown pathology	Protects neurons against oxidative stress; some evidence for a role in autophagy	[117]
*ATP13A2*	AR	Juvenile-onset atypical parkinsonism (Kufor–Rakeb); pathology demonstrates ceroid lipofuscinosis	Encodes a lysosomal membrane transporter; mutations cause lysosome dysfunction and increased exosomal secretion of α-synuclein.	106, 118
*PLA2G6*	AR	Juvenile-onset atypical parkinsonism with brain iron accumulation	Encodes a calcium-independent phospholipase, which as a group have roles in membrane trafficking.	[119]
*FBX07*	AR	Juvenile-onset atypical parkinsonism with unknown pathology	Component of E3 ubiquitin protein ligases that participate in phosphorylation-dependent ubiquitination	[120]
*DNAJC6*	AR	Juvenile-onset atypical parkinsonism with unknown pathology	Involved in clathrin-mediated Golgi–lysosome trafficking and synaptic vesicle endocytosis.	93, 94
*SYNJ1*	AR	Juvenile-onset atypical parkinsonism with unknown pathology	Encodes synaptojanin-1, a presynaptically enriched protein involved in synaptic vesicle exocytosis	[91]

a AD, autosomal dominant; AR, autosomal recessive.

## Box 1 The pathological and physiological species of α-synuclein remain controversial

In 1912 the characteristic neuropathological triad of PD was first described: neuronal loss in the substantia nigra and, in the remaining neurons, two abnormal proteinaceous aggregates termed Lewy bodies and Lewy neurites [110]. It was not until 1997 that the predominant component of Lewy pathology was discovered to be α-synuclein, an evolutionarily conserved 140 amino acid presynaptic protein [7]. α-Synuclein is neurotoxic in insoluble aggregated states such as fibrils, but, as in other neurodegenerative diseases, there is increasing evidence that soluble oligomeric species contribute most to toxicity. Duplications and triplications in the α-synuclein gene, *SNCA*, cause PD, as do point mutations, the most common being Ala30Pro, Gly46Lys, and Ala53Thr (Figure I). Despite advances in knowledge of disease pathogenesis, the true physiological species of α-synuclein remains enigmatic. α-Synuclein does not assume a predictable structure in aqueous solution, and thus has been historically described as an intrinsically disordered monomer of approximately 14 kDa. Recent experimental work has attempted to shed new light on the physiological form of α-synuclein. In 2011, Bartels *et al.* used native polyacrylamide gel electrophoresis (native-PAGE), rather than more conventional detergent-based PAGE, to avoid denaturation of physiological α-synuclein assemblies, and analysed human cell lines, red blood cells (RBC), and mouse cortex [111]. In addition to multiple other methods, they developed a novel technique of purifying α-synuclein from RBC lysates and examined isolated α-synuclein by electron microscopy. In all these studies a predominant endogenous species of 55–60 kDa was detected, corresponding to a presumed α-synuclein tetramer, which Bartels and coauthors put forward as the primary physiological species. They suggested that α-synuclein monomers may be prone to aggregation, and that stabilization of tetrameric α-synuclein could be a useful therapeutic strategy. The conclusions of this study were disputed by Burré and colleagues in 2013 [112] who acknowledged that, although native and recombinant α-synuclein migrated at approximately 55 kDa on native-PAGE, the same migration pattern was apparent after disrupting secondary and tertiary structures by boiling protein samples. They determined that the bulky unstructured state of monomeric α-synuclein explained the apparent increase in molecular mass on native-PAGE and hydrodynamic radius on electron microscopy. In addition, Burré *et al.* purified native mouse brain α-synuclein and subjected it to mass spectrometry, which yielded a mass of approximately 16 kDa – consistent with an acetylated α-synuclein monomer. Further investigations have been published alternatively supporting either monomeric [113] or tetrameric [114] α-synuclein as the predominant physiological species. Further work should focus on α-synuclein dynamics in iPSC DA neurons, which provide an *in vitro* avenue for interrogating human PD pathophysiology.

## Box 2 Outstanding questions

• What is the predominant physiological species of α-synuclein in healthy human and PD patient brain and in iPSC-derived dopaminergic neurons?
• How old do iPSCs need to be to recapitulate age-related disease phenotypes?
• What is the effect of the chromosome 17q21 H1 and H2 haplotypes on NSF function?
• What are the neurobiological differences that cause substantia nigra dopaminergic neurons to be more susceptible in PD than their counterparts in the ventral tegmental area?
• Can *in vivo* imaging of striatal synapses in PD models demonstrate early events in synaptic dystrophy?
• Can novel therapeutic agents that aid intracellular trafficking ameliorate PD?
• 3D synapse modelling has recently been developed. Can this be used to define the early synaptic changes in PD?

## Glossary

α-Synuclein: a presynaptically enriched protein that acts in conjunction with SNARE proteins to regulate neurotransmitter release. It is the major component of two pathological hallmarks of PD: proteinaceous aggregates termed Lewy bodies and Lewy neurites. α-Synuclein is encoded by the gene *SNCA*.
Dopamine (DA): a neurotransmitter derived from the amino acid tyrosine. It is released by DA neurons of the midbrain onto medium spiny neurons in the striatum to help regulate movement.
Glucocerebrosidase (GBA): an enzyme that cleaves glucocerebroside, an intermediate protein in glycolipid metabolism. It is encoded by the gene *GBA*. Patients who suffer from Gaucher's disease, a lysosomal storage disease, are homozygous for mutations in *GBA*. A subset of patients suffering Gaucher's disease exhibit parkinsonian symptoms. Patients who are *GBA* mutation heterozygotes have an increased risk of developing PD.
iPSCs: mutipotential cells derived from differentiated adult cells that can be manipulated to produce different cell types including neurons.
Leucine rich repeat kinase 2 (LRRK2): a large multidomain protein with GTPase and kinase domains that has multiple intracellular roles, including regulating autophagy. It is encoded by the gene *LRRK2*. Mutations in *LRRK2* cause familial PD, and polymorphisms in *LRRK2* confer risk of sporadic PD.
Mesostriatal pathway: the neural pathway that connects the midbrain to the striatum. DA-producing neurons whose cell bodies and dendrites reside in the midbrain, and in particular the substantia nigra, make synaptic connections within the striatum.
SNARE (soluble *N*-ethylmaleimide–sensitive factor attachment protein receptor) proteins: form complexes which mediate docking of neurotransmitter-containing vesicles to the presynaptic membrane. Important SNARE proteins include synaptobrevin, syntaxin, and SNAP-25.
Substantia nigra: literally ‘black substance’, a region of the midbrain where pigmented neuromelanin-containing dopaminergic neurons are located. It is divided into two main parts: the pars compacta (SNc) and pars reticulata. Loss of neurons from the SNc is one the pathological hallmarks of PD. Lewy bodies and neurites are present in some of the remaining neurons in most cases of PD.
Tau: a microtubule-associated protein that is present in the axons of neurons, and assists and helps to regulates axonal transport. Tau is encoded by the gene *MAPT*.
